# 3D Bioprinted GelMA/PEGDA Hybrid Scaffold for Establishing an In Vitro Model of Melanoma

**DOI:** 10.4014/jmb.2111.11003

**Published:** 2022-01-13

**Authors:** Jiahui Duan, Yanyan Cao, Zhizhong Shen, Yongqiang Cheng, Zhuwei Ma, Lijing Wang, Yating Zhang, Yuchuan An, Shengbo Sang

**Affiliations:** 1 MicroNano System Research Center, College of Information and Computer and Key Lab of Advanced Transducers and Intelligent Control System of the Ministry of Education, Taiyuan University of Technology, Taiyuan 030024, P.R. China; 2 College of Information Science and Engineering, Hebei North University, Zhangjiakou 075000, P.R. China

**Keywords:** A375 cells, GelMA/PEGDA, luteolin, melanoma, in vitro, 3D bioprinting

## Abstract

Due to the high incidence of malignant melanoma, the establishment of in vitro models that recapitulate the tumor microenvironment is of great biological and clinical importance for tumor treatment and drug research. In this study, 3D printing technology was used to prepare GelMA/PEGDA composite scaffolds that mimic the microenvironment of human malignant melanoma cell (A375) growth and construct in vitro melanoma micro-models. The GelMA/PEGDA hybrid scaffold was tested by the mechanical property, cell live/dead assay, cell proliferation assay, cytoskeleton staining and drug loading assay. The growth of tumor cells in two- and three-dimensional culture systems and the anti-cancer effect of luteolin were evaluated using the live/dead staining method and the Cell Counting Kit-8 (CCK-8) method. The results showed a high aggregation of tumor cells on the 3D scaffold, which was suitable for long-term culture. Cytoskeleton staining and immunofluorescent protein staining were used to evaluate the degree of differentiation of tumor cells under 2D and 3D culture systems. The results indicated that 3D bioprinted scaffolds were more suitable for tumor cell expansion and differentiation, and the tumor cells were more aggressive. In addition, luteolin was time- and dose-dependent on tumor cells, and tumor cells in the 3D culture system were more resistant to the drug.

## Introduction

According to the journal *Cancer Statistics* 2021, malignant melanoma is the most common form of cancer. Melanoma is one of the ten most common cancers in both men and women, accounting for 6% of cases in males and 5% in females. New cases of melanoma in the United States are expected to reach approximately 110,000 in 2021 [1]. At the present, the treatment of melanoma mainly includes surgical resection, immunotherapy and targeted therapy [2]. However, the usefulness of these treatments is limited due to poor sensitivity, side effects, and drug resistance, and new, more effective methods need to be explored to cope with the progressive development of melanoma [3]. Currently, natural products, especially traditional herbal medicines, are diffusely accepted in Western countries as new adjuvant medicines to inhibit the spread of cancer and prolong the survival of patients [4]. Traditional Chinese herbs exert anti-cancer effects by inducing apoptosis and inhibiting differentiation and angiogenesis, enhancing immunity, and reversing Multiple Drug Resistance (MDR) [5]. Luteolin is a natural flavonoid found in plants such as honeysuckle and perilla, and possesses various pharmacological properties with anti-inflammatory, anti-allergic, anti-tumor and anti-bacterial effects [6]. In recent years, luteolin has been shown to inhibit tumor metastasis and induce apoptosis in tumor cells [7]. Luteolin has also been reported to have significant inhibitory effects on solid tumors such as hepatocellular carcinoma (HepG2 cells), breast cancer (MCF-7 cells), cervical cancer (Hela cells) and prostate cancer (LNCaP cells) [8-11]. While luteolin has an effective inhibitory effect on certain cancer cells, it is rarely reported whether it can inhibit the proliferation and spread of A375 cells.

General 2D culture cell biology studies provide a simple, low-cost approach, but the tumor microenvironment is a dynamic cell population that has a variety of chemical or biological and significant spatial gradient signatures [12, 13]. In vitro cell culture-based 3D bioprinting in cancer research is a new and creative approach to reducing the differences with conventional 2D-cultured tumors in vivo [14]. The cellular and ECM components in the microenvironment of this 3D culture system can be precisely controlled [15]. The scaffold provides a tissue-specific structure that affords the necessary support for cell proliferation, migration, differentiation, ECM deposition, and response to stimuli similar to in vivo biological systems and may reduce some of the differences between in vitro and in vivo outcomes [16]. Currently, hydrogels are considered suitable materials for the biological field, while gelatin is composed of a matrix metalloproteinase (MMP) target pattern formed by hydrolysis of mammalian collagen [17]. Gelatin methacryloyl (GelMA) has become one of the most versatile biomaterials. It was originally proposed by Van Den Bulcke *et al*. in 2000 [18]. Polyethylene (glycol) diacrylate (PEGDA) is a hydrogel with good hydrophilicity, and its contact angle is 70° at a concentration of 1.25% [19,20]. Moreover, its elasticity is also very good, and its compressive modulus can reach 0.75 MPa at a concentration of 30% [21]. It can gel rapidly at room temperature in the presence of blue light and photoinitiators. The mechanical properties of PEGDA can vary over a wide range, and when it is mixed with GelMA, the hydrophilicity and mechanical strength of the hydrogel can be greatly improved [22]. GelMA/PEGDA hybrid hydrogel has the characteristics of good mechanical strength, high printing resolution, and resistance to degradation, and has bioactive sequences for cell attachment and matrix metalloproteinase (MMP)-sensitive degradation sites [22, 23]. Based on these properties, the interaction between in vivo cells and in vitro matrix (ECM) can be simulated by an in vitro 3D model established by GelMA and PEGDA.

In this study, GelMA/PEGDA hydrogel scaffolds mimicking the tumor cell growth microenvironment were further prepared by 3D bioprinting with appropriate concentrations of GelMA and PEGDA as materials. Then, the behavior of A375 cells in both 3D and 2D culture systems was compared. In addition, the inhibitory effect of luteolin on A375 cells on 2D and 3D scaffolds was discussed and differences in drug effects were found.

## Materials and Methods

### Materials and Reagents

Dulbeccós modified Eagle medium (DMEM), fetal bovine serum (FBS), horse serum (HS), trypsin-EDTA and phosphate-buffered saline (PBS) were purchased from Gibco (Singapore). A375 cells were purchased from the Cell Bank of the Chinese Academy of Sciences.

### Major Research Processes

**Fig. 1:**
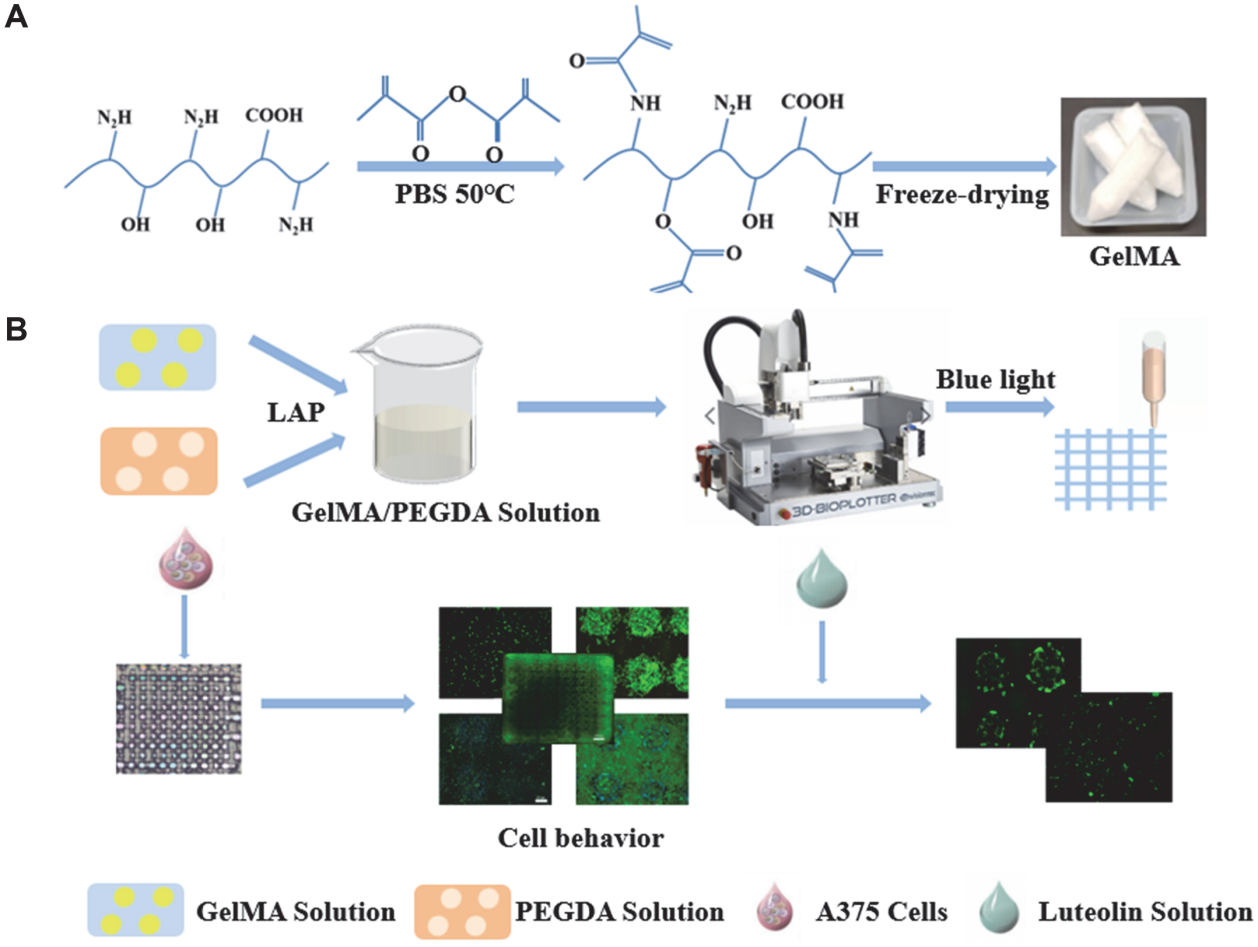
Preparation and characterization of GelMA/PEGDA 3D bioprinted scaffolds and establishment of in vitro models of melanoma (A) Preparation of GelMA. (B) Establishment of A375 cell culture model in vitro.

Fig. 1 shows the preparation process for the 3D GelMA/PEGDA scaffold and the process of exploring the antitumor effect of luteolin on A375 cells. First, composite scaffolds were prepared using 3D bioprinting technology and tumor cells were cultured in vitro for greater precision in the drug screening. The processes primarily included the synthesis of GelMA (Fig. 1A), selection of different concentrations of ink, adjustment of printing performance, bioprinting, cross-linking, and scaffold handling. Subsequently, the scaffolds were gently rinsed several times with ultrapure water and sterilized under UV light for future cell culture. We then constructed a 3D tumor model with 3D bioprinted composite scaffolds and tested the anticancer effect of luteolin and the growth of tumor cells in different culture systems in both 2D and 3D culture systems (Fig. 1B).

### Preparation of GelMA

First, 100 ml of PBS buffer was heated to 50°C in a water bath. Then, 10 g of gelatin was weighed into the PBS at 50°C and stirred until completely dissolved. Following that, 8 ml of methacrylamide was slowly added to the above dissolved solution in 16 portions at a rate of 0.5 ml/min. The reaction took place in a water bath at 50°C for 2 h. Next, 400 ml of PBS buffer was added to dilute the solution, which was then stirred for 10 min. The diluted solution was poured into the co-dialysis membrane and tied tightly. The dialysis membrane was then submerged in a beaker filled with ultrapure water and dialyzed in a 40°C water bath for 5-7 days. The solution was changed three times a day and shaken well. At the end of dialysis, the supernatant was collected by centrifuge and freeze-dried for 48 h to obtain GelMA solid [24].

### Characterization of Materials

**Preparation of GelMA/PEGDA scaffold**. Solutions of GelMA/PGEDA with mass fractions of 2.5/2.5%, 5/ 2.5%, 7.5/2.5%, 10/2.5%, 12.5/2.5% (w/v) were prepared by dissolving GelMA and PEGDA in PBS buffer. The mixed solutions were cured and cross-linked under blue light to form a cylinder with a radius of 8 mm and a height of 5 mm.

**Young's modulus test.** Young's modulus of different scaffolds was evaluated by using a uniaxial compression tester (Norwood 5556 Research Institute, USA). Prior to testing Young's modulus of the scaffolds, the dry scaffolds were soaked in PBS for 24 h. The scaffolds were then removed and excess water was gently wiped from the surface with filter paper. The initial cross-sectional area and thickness of each sample were then measured. The next step was to slowly apply a preload force to the samples. The compression rate was set to 0.1 mm/min. When the scaffold was compressed to about 50% of its original length, the modulus of compression was calculated based on the slope of the initial linear region of the stress-strain curve.

**Water absorption test**. Three parallel samples were provided for each of the cylindrical scaffolds of different concentrations. The samples were placed in Petri dishes of known mass and 4 ml of PBS solution was added to each and soaked at 37°C for 24 h to reach the swelling equilibrium. The PBS was aspirated, the remaining water droplets were wiped off with filter paper, and the swollen sample masses (W_s_) were weighed. All samples were freeze-dried and weighed to obtain their dry weight (W_d_). The swelling rate (Q_s_) of the samples was calculated according to the following equation:

$$Q_{S}(\%)=(W_{S}-W_{d})/W_{d}.$$ (1)

Porosity. Porosity was measured by the drainage method. The radius (r) and height (h) of each lyophilized scaffold were measured and weighed (M1). The scaffolds were then immersed in absolute ethanol and placed in a vacuum drying oven with reduced pressure to remove air bubbles. After 3 h of immersion, the surface water was filtered off with filter paper and then weighed on an electronic balance (M1 for the weight before immersion and M2 for the weight after immersion). The porosity (θ) is calculated by the following formula (ρ is the density of ethanol 0.789 mg/ml):

$$\theta =\frac{M_{2}-M_{1}}{\frac{\rho }{\pi r^{2}h}}.$$ (2)

**Scanning electron microscopy (SEM) analysis**. The lyophilized scaffolds were obtained after lyophilization, and the scaffolds were cut along the cross section with a scalpel and sprayed with gold. The microstructure of the lyophilized scaffolds was observed by SEM (JEOL, Japan) at an accelerating voltage of 5V.

**Cytotoxicity test**. Live/dead cell staining experiments were performed to observe the viability of A375 cells cultured in GelMA/PEGDA complex gels at different concentrations. Live and dead cells were double-stained with calcein-AM and propidium iodide (PI). Calcein-AM is a cell-staining reagent that fluorescently labels live cells. When it enters the cytoplasm, esterases hydrolyze it to calcein and leave it inside the cell, emitting green fluorescence. PI reaches the nucleus only through the disordered region of the dead cell membrane and is embedded in the cell's DNA double helix structure, producing red fluorescence. The above hydrogel solutions at different concentrations were first added to 24-well plates at 0.5 ml/well, solidified with blue light, and then inoculated with 100 μl of cell suspension (5 × 10^6^ cells/ml) and incubated in a 37°C, 5% CO_2_ incubator for 24 h. After 1 day of culture, calcein-AM/PI double-stained working solutions were prepared and incubated in a humidified incubator for 45 min. Then, the dye solutions were aspirated and the samples were washed three times with phosphate-buffered saline (PBS). Morphological photographs of the stained cells were taken with a Cytation 5 Imaging Reader (BioTek, USA). The number of live and dead cells was analyzed from the fluorescent images.

### 3D Bioprinting

GelMA and PEGDA were used as materials to prepare 3D bioprinted scaffolds to build in vitro tumor models. The 3D printing ink was prepared from GelMA and PEGDA in a 4:1 by mass ratio to obtain a 10/2.5% GelMA/PEGDA mixture. The 3D scaffolds were designed using computer-aided design software. The length, width, and height of the 3D scaffold were 10 mm, 10 mm, and 1.2 mm, respectively. The spacing of 3D supports is 0.8mm and the number of layers is 6. The 3D scaffold was then printed by an extrusion printing technique using a 3D bioprinter (EnvisionTEC GmbH, Germany). The radius of the printing needle was 0.08 mm, the print speed was set to 12.5 mm/s, the pressure was 1.7 bar, and the temperature was set to 17°C. After printing, the scaffold was shaped by irradiating it with blue light for 1 min. Then, after the scaffolds were completely cross-linked, they were sterilized under a UV sterilization lamp for 24 h. Finally, the scaffolds were gently rinsed several times with deionized water and stored for subsequent cell experiments.

### Cell Culture

A375 cells were cultured in DMEM containing 10% FBS and 1% penicillin-streptomycin in a humidified incubator at 37°C and 5% CO_2_. When the cells covered 80-90% of the bottom of the culture flask, a passage experiment was performed to ensure viability for subsequent usage of the cells.

### Cell Growth Activity Assay

The activity of A375 cells in 2D and 3D scaffolds was detected by the live/dead staining method and CCK-8 method, respectively. First, 3D printed scaffolds were inoculated with 100 μl of cell suspension (2 × 10^6^ cells/ml). Cell scaffold complexes were placed on 12-well plates. After 1, 3, 5, and 7 days of incubation, the cells were stained with a staining solution consisting of 2 μmol/l calcein-AM and 4 μmol/l PI and incubated at 37°C with 5% CO_2_ for 45 min. The dye solution was then removed with a pipette and the samples were washed three times with phosphate-buffered saline (PBS). An enzyme marker was used to detect cell viability. In the 2D scaffold, 540 μl of mixed hydrogel solution was added to each well of a 12-well plate for UV-cured cross-linking. The same number of cells were then inoculated in the 12-well plates and cultured for 1, 3, 5, and 7 days and assayed as described above. Considering the effective range of OD values, 5,000 cells were inoculated in 2D and 3D scaffolds when performing the CCK-8 analysis. After 1, 3, 5, and 7 days of cell culture, 100 μl of fresh medium was replaced with 10 μl of CCK-8 working solution and incubated in an incubator for 40 min. Then,100 μl of the test solution was added dropwise to a 96-well plate and the absorbance was measured at 450 nm with a time of 10 s. Cell proliferation and activity were compared in 2D and 3D cultures.

### Phalloidin/DAPI Staining

To assess the growth of tumor cells on 2D and 3D scaffolds, a 100 μl A375 cell suspension (2 × 10^6^ cells/ml) was inoculated on the scaffolds and incubated for 12 h. The culture medium was removed and washed 3 times with sterile PBS. The medium was then incubated in an incubator for 5 days using DMEM medium containing 10%FBS. The differentiated cell scaffolds were fixed with 4% paraformaldehyde (PFA) solution and infiltrated with 0.3% Triton X-100 solution. Cells attached to the scaffold were stained with phalloidin and fluorescence images were acquired using the Cytation 5 Imaging Reader (Bio-Tek).

### Immunofluorescence Staining

After 5 days of differentiation culture, the scaffolds were washed 3 times with PBS. The scaffolds were fixed with 4% PFA for 15 min, then permeabilized with 0.5% Triton X-100 (PBS preparation) for 20 min at room temperature, washed with PBS, aspirated, and closed with normal goat serum for 30 min at room temperature. Then, the closure solution was aspirated, a sufficient amount of diluted primary antibody was added dropwise, and the solution was left overnight at 4°C. The next day, the cell scaffold was reacted with a secondary antibody dilution of goat anti-rabbit IgG antibody-Alexa Fluor 546 coupling (1:200, Invitrogen) for 30 min and incubated with DAPI solution for 15 min at room temperature. Fluorescent images were obtained using the Cytation 5 reader (Bio-Tek).

### Cell Activity Assay After Exposure to Luteolin

Live/dead staining and CCK-8 analysis were used to assess the impact of different concentrations of luteolin on A375 cell growth. Luteolin was dissolved in the culture medium at different concentrations and decontaminated with a filter membrane (pore size 0.22 μm). Then, 2 × 10^5^ A375 cells were inoculated on 2D stents and 3D printed scaffolds and cultured for 5 days to form tumor cell clusters. They were then exposed to different concentrations of luteolin for 24 and 48 h and cell viability was assessed by CCK-8 and live/dead staining assays.

### Statistical Analysis

All data are expressed as mean ± standard deviation (SD). One-way analysis of variance (ANOVA) was used for significance analysis. *p* < 0.05 was considered a significant difference. OriginPro2018 software (Origin Lab, USA) was used for all statistical analyses.

## Results and Discussion

### Characterization of GelMA/PEGDA Scaffolds

Structural support for cell growth is provided by GelMA [25]. Pure gelatin lacks mechanical strength, and the shrinkage of gelatin during the manufacturing process destroys the microstructure. On the other hand, natural gelatin comes with the risk of immunogenicity and pathogen transmission. Gelatin methacrylate (GelMA) is prepared from methacrylic anhydride (MA) and gelatin (gelatin). It is a photosensitive biohydrogel material with good biocompatibility. It can generate a solidification reaction under the excitation of blue light to form a three-dimensional structure with certain strength suitable for cell growth and differentiation. The biocompatibility of GelMA is much better than that of Matrigel and Fibrin Glue, and its properties are similar to collagen. At the same time, its molding properties are much better than that of collagen, making it the best choice to replace these other materials. Polyethylene glycol (ethylene glycol) diacrylate (PEGDA) hydrogels are considered bioinert, and their mechanical properties can vary over a wide range of moduli. GelMA/PEGDA hybrid hydrogel has the characteristics of good mechanical strengths, high printing resolution, and resistance to degradation. Tumor models based on 3D printing can provide cell adhesion sites, which can change the gene expression and signal transmission of tumor cells to a certain extent. In addition, the bioactive sequences for cell attachment and matrix metalloproteinase (MMP)-sensitive degradation sites are retained in the gelatin skeleton [23], which helps to research melanoma cell invasion and aggregation [26]. Furthermore, the low molecular weight of PEGDA gives it more flexibility in the reaction system. PEGDA is a bifunctional monomer that increases the number of unsaturated double bonds in the system and improves the degree of cross-linking of the hydrogel. The pure GelMA scaffold is not suitable for long-term culture of melanoma cells with rapid proliferation in a culture environment of 37°C and 5% CO_2_. Therefore, the addition of PEGDA keeps the hydrogel stable for a long time at 37°C. Both natural materials are extensively used to construct a 3D scaffold in a cell culture system. Even more encouraging, the blend of GelMA and PEGDA has also been reported to improve the bioactivity and mechanical properties of hydrogels, and due to the hydrophilic nature of PEGDA, it also helps to improve hydrophilicity as well as oxygen and nutrient exchange [23]. Considering these factors, GelMA and PEGDA are suitable materials for the construction of 3D scaffolds.

**Fig. 2:**
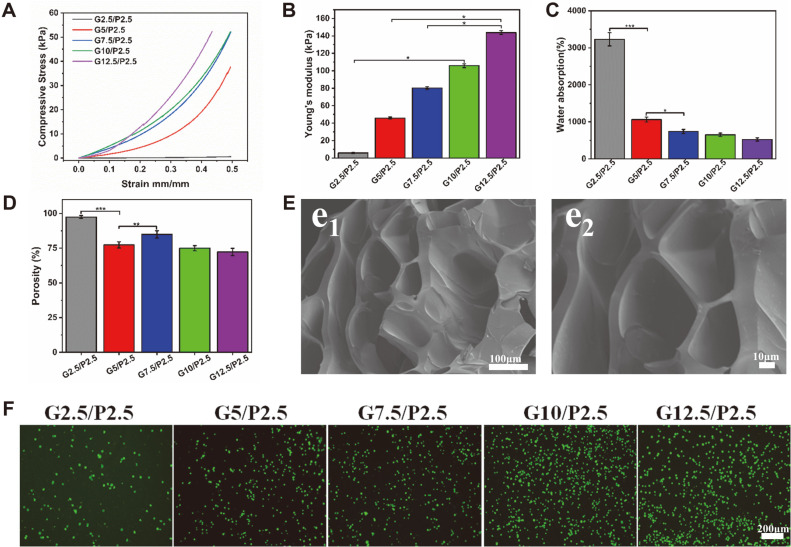
Detection of GelMA/PEGDA composite scaffold. (**A**) Compressive stress curve of composite bracket (*n* = 3). (**B**)Young's modulus of composite scaffold (*n* = 3). (**C, D**) Water absorption and porosity of scaffolds (*n* = 3). (**E**) SEM micrographs of the lyophilized GelMA10%/PEGDA2.5%. (e1)200×. (e2)400×. (**F**) Cytotoxicity test of different concentrations of scaffolds. ***p < 0.001, **p < 0.01, *p < 0.05.

To prepare 3D hydrogel scaffolds, the GelMA/PEGDA hydrogels were made into a cylindrical solid by a UV light-curing technique. Then they are freeze-dried to obtain lyophilized scaffolds. The cross-section of the hydrogels (Fig. 2E) exhibits a loose, porous morphology, which facilitates the uptake of oxygen and nutrients. The pore size of the hydrogel material is estimated to be 129 ± 13 μm. Pore sizes that are too narrow may limit cell proliferation, but scaffolds with pore sizes that are too wide will decrease the surface area for cell adherence. This lattice structure may enhance the mechanical properties of the material and reduce the risk of scaffold lysis. In addition, this interoperability of pores conveniently provides for better nutrient exchange and cell migration [27]. In general, 3D printing ink has excellent mechanical properties and can support tumor cells in culture for long periods. The results of mechanical performance tests showed that the stress on the scaffold of the GelMA/PEGDA hybrid hydrogel becomes higher with the increase of GelMA concentration (Fig. 2A). Taking the hydrogel with G10/P2.5 concentration as an example, the stresses of the scaffold reached 5.0311 kPa and 52.210 kPa at strains of 0.1 and 0.5, respectively, indicating that the scaffold has good compressive properties. The Young's modulus also revealed the same tendency as the compressive stress curve (Fig. 2B). In addition, the swelling rate and porosity of the hybrid hydrogel were tested. As shown in Fig. 2C, the results indicated that the hybrid hydrogel has good hydrophilicity and the water absorption performance of the material will slightly decrease with increasing GelMA concentration. The water absorption and porosity of the G10/P2.5 scaffold were 649.54 ± 45.60% and 74.93 ± 1.87% (Fig. 2D). The performance tests of mixed hydrogels at different concentrations showed that GelMA/PEGDA could be applied to cellular experiments.

Based on these good physical properties, we further tested the biocompatibility of the hybrid hydrogel material. According to Fig. 2F, the number of A375 cells was relatively low on the hydrogel at a concentration of G2.5/P2.5. The reason may be that the hydrogels were too soft for the cells to adhere well and were washed away when washed with PBS [28]. The amount of cells in the G5/P2.5 group was relatively high, but there was still a problem with cell attachment. With the increase of GelMA concentration, the mixed hydrogel A375 cells could adhere well at the three concentrations of G7.5/P2.5, G10/P2.5 and G12.5/P2.5. The results of live/dead staining experiments showed that a large number of cells survived, indicating that the mixed hydrogel material with this concentration has good biocompatibility. Based on this, the well-performing G10/P2.5 concentration was used as a follow-up experiment for 3D bioprinting.

### 2D and 3D Cell Culture

**Fig. 3:**
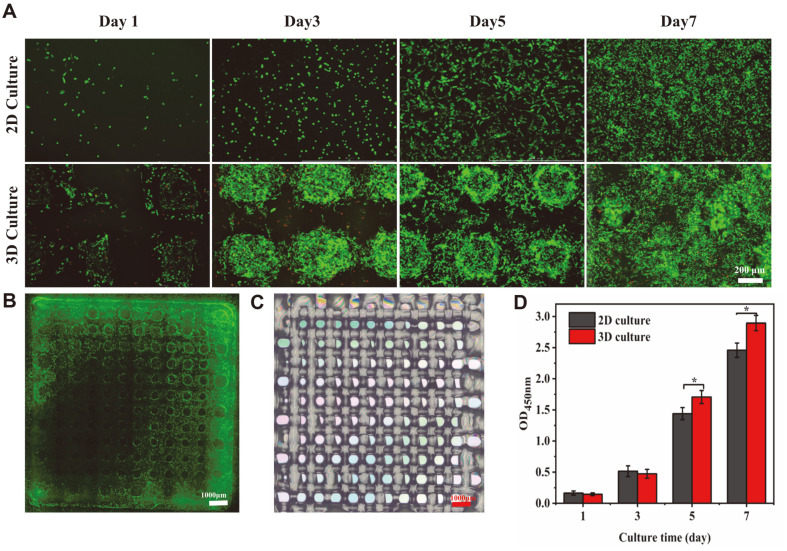
Activity of A375 cells on scaffolds. (**A**) Cell activity was assessed by live/dead staining after 1, 3, 5 and 7 days of culture. (**B**) Panorama of live/dead stained 3D scaffold (**C**) Macro view of 3D bioprinted scaffold. (**D**) 12-well plates and 3D scaffolds were used to assess A375 cell activity with CCK-8 after 1, 3, 5 and 7 days of culture (*n* = 3). ***p < 0.001, **p < 0.01, *p < 0.05.

Excellent cytoactive is a key requirement for the construction of in vitro tumor models. Live/dead staining and CCK-8 assays were used to explore the liveness of A375 cells on the scaffold (Fig. 3B). As shown in Fig. 3A, most living cells were stained with calcein-AM on different days and expressed strong green fluorescence. The green fluorescence was significantly enhanced as the incubation time increased, indicating that A375 cells kept up high liveness properties on the scaffold and the amount of dead cells stained by PI showed a slight upward trend. The main causes of cell death were insufficient nutrition and oxygen supply [29]. The staining results also showed that as the number of days of cell culture increased, the cells continued to proliferate until they covered the entire 3D scaffold. After 7 days of culture, the cells on the 3D scaffold could grow in aggregates on the scaffold, showing spherical cell clusters due to the structural characteristics and good biocompatibility of the 3D bioprinted scaffold. Compared with 2D scaffolds, 3D scaffolds could better promote the aggregation and growth of tumor cells. CCK-8 assays were used to detect and compare the proliferation of 2D and 3D scaffold-cultured cells (Fig. 3D). Furthermore, the results of calcein-AM and PI staining as well obviously indicated that the cells continued to proliferate during 7 days of culture. On day 1, cell proliferation was lower in the 3D scaffold than in the 2D scaffold. The difference in cell numbers on day 3 gradually decreased as the incubation time increased. From the fifth day, cell proliferation on the 3D scaffolds exceeded that on the 2D scaffolds. With fewer culturing days, the proliferation of cells on 2D scaffolds was higher than that of cells on 3D scaffolds. The reason may be related to the adaptability of the cells. For a short period, cells are more likely to attach and proliferate on a flat surface. When the culture time is longer, as the cells keep proliferating, the 2D scaffold will show cell contact inhibition faster than the 3D scaffold, while the 3D scaffold's three-dimensional structure can provide a larger growth area for the cells, and thus the cells can proliferate more.

### Phalloidin /DAPI Staining

**Fig. 4:**
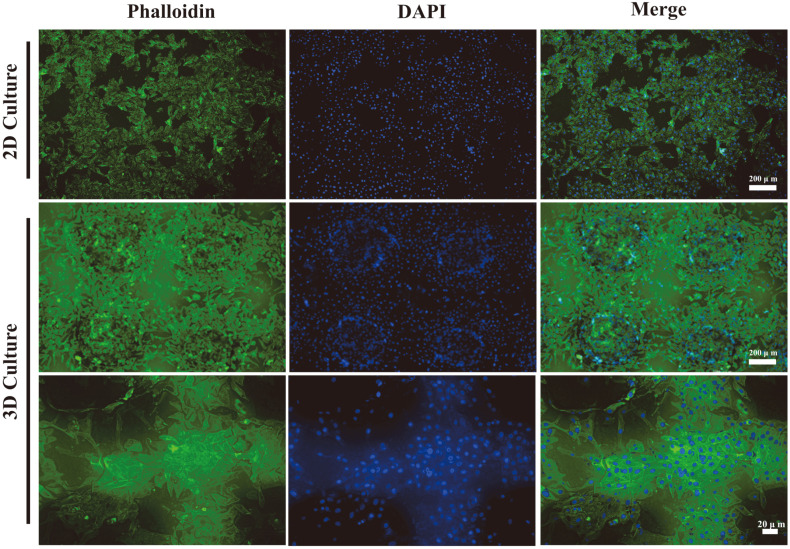
Cytoskeleton staining to observe the growth of A375 cells on the scaffold (scale bar = 200 μm).

After 5 days of inoculation culture, the 2D-cultured and 3D-cultured A375 cells were stained with FITC-labeled phalloidin and their specific morphology was observed under a microplate reader . As shown in Fig. 4, the 2D-cultured A375 cells were laid flat on a flat surface, and the staining image on the 3D scaffold showed that A375 cells spread around the scaffold to the edge, the cells in the middle part aggregated into clusters, and the A375 cells were more elongated in shape. DAPI staining results showed that the 3D scaffold had a different height spatial distribution of cells compared to the 2D scaffold, which was closer to the human environment. In different culture systems, the growth morphology of the cells on the 2D scaffold and the 3D-printed scaffold was found to be different [30]. In the 3D culture system, the 3D-cultured A375 cells showed stronger aggregation and migration. The results suggested that A375 cells clustered more easily and dispersed more fully in the 3D culture system.

### Immunofluorescence Staining

**Fig. 5:**
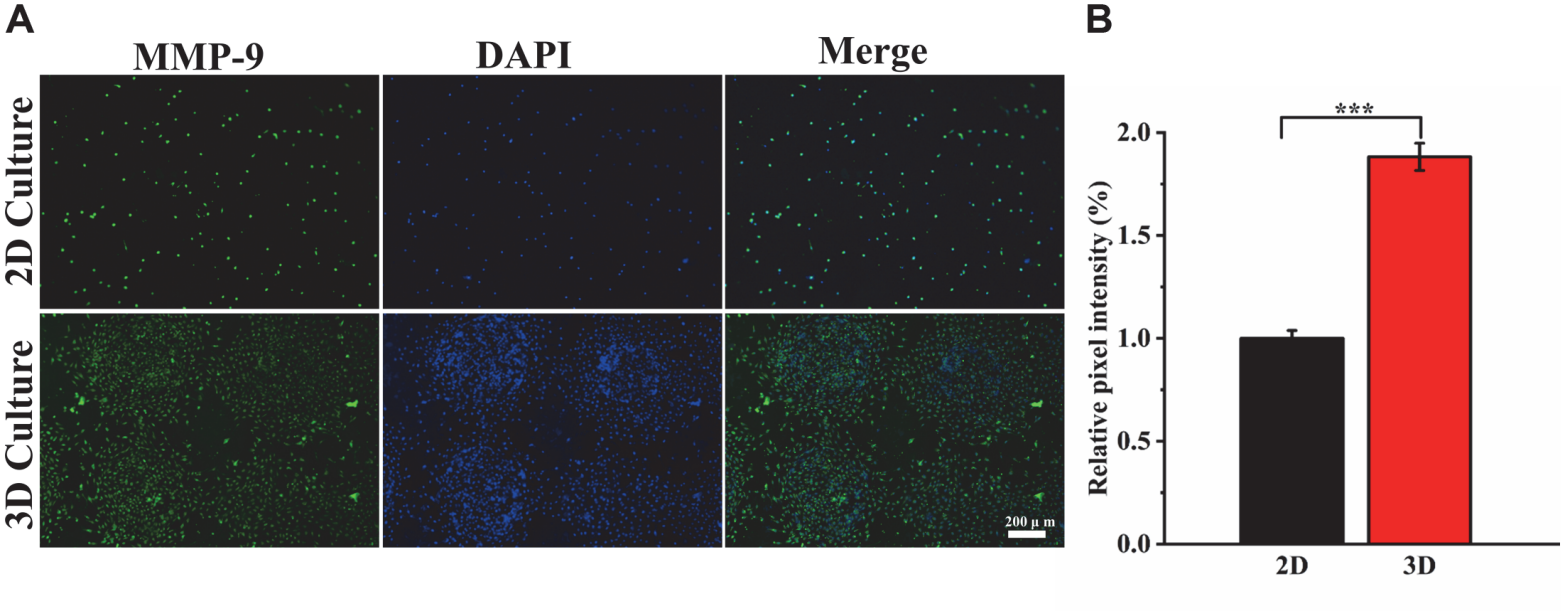
Immunofluorescence staining chart. (**A**) MMP-9 protein expression of A375 cells on scaffolds was evaluated by immunofluorescence staining after 4 days of differentiation culture (scale bar = 200 μm). (**B**) Relative intensity of immunofluorescence staining. ***p < 0.001, **p < 0.01, *p < 0.05.

Immunofluorescence staining experiments further verified the effect of different culture systems of 2D scaffolds and 3D printed scaffolds on the differentiation ability of A375 cells. MMP-9 is a matrix metalloproteinase whose main function is to degrade and remodel the extracellular matrix [31]. In addition to the prototypical structure of MMPs, the catalytic region of MMP-9 includes three repetitive fibronectin structural domains. This structural domain has a high affinity for gelatin, which fits well with the material used in our scaffold. In addition, MMP-9 can be involved in angiogenesis through the release of vascular endothelial growth factor (VEGF) [32]. It is well known that blood vessels play a very important role in tumor formation. Green fluorescence-labeled secondary antibodies were used to detect MMP-9 protein. Moreover, DAPI was used for staining to determine the location of the cell nucleus. The stronger the green fluorescence signal, the higher the differentiation of the surface cell protein. As shown in Fig. 5A, cells on 2D and 3D scaffolds have differentiated to express MMP-9 protein, and the intensity of the green fluorescence signal was significantly different in different culture systems. In addition, semi-quantitative analysis of the images using ImageJ software showed that the relative fluorescence intensity of the 3D scaffolds increased by 88.24 ± 6.61% compared to the 2D scaffolds (Fig. 5B). The fluorescence area on the 3D scaffold was larger and the MMP-9 protein expression level of A375 cells was higher compared to the 2D scaffold. The results suggest that A375 cells are more aggressive in the 3D culture system and more closely resemble tumor growth in vivo.

### Effect of Luteolin on A375 Cell Apoptosis

**Fig. 6:**
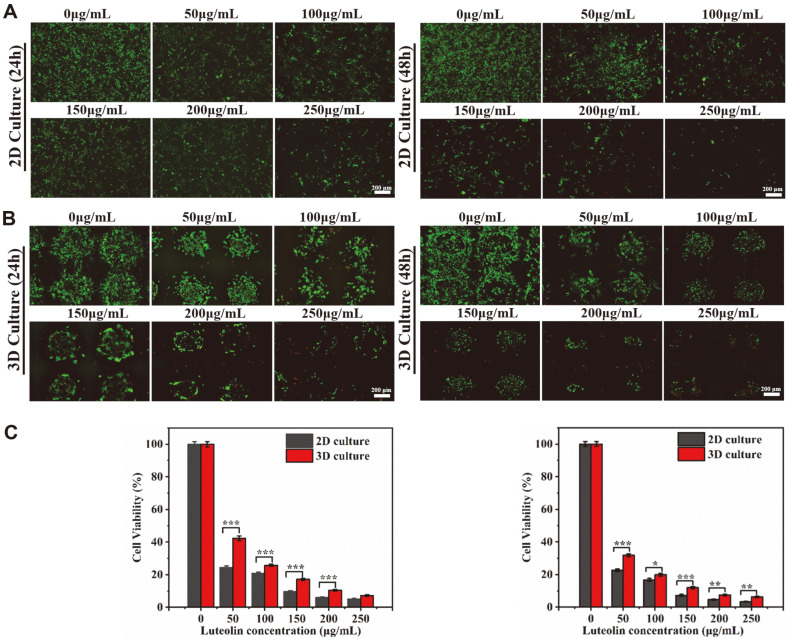
Inhibitory effect of luteolin on A375 cells in 12-well plates and 3D scaffolds. (**A, B**) Cells were stained with calcein-AM and PI after 24 and 48 h of exposure to luteolin (scale bar = 200 μm). (**C**) Survival of A375 cells exposed to luteolin was assessed by CCK-8 assay after 24 and 48 h of culture, respectively (*n* = 3). ***p < 0.001, **p < 0.01, *p < 0.05.

To test the inhibitory effect of luteolin on tumor cells on 2D and 3D scaffolds, A375 cells were seeded at a high density and cultured for 5 days to form a cell mass (Figs. 6A and 6B). After 5 days of culture, luteolin at gradient concentrations of 0-250 μg/ml was added and incubated for 24 and 48 h. Live/dead stained images clearly show the growth of a cell mass on the scaffolds. After the undosed group continued to be cultured for 2 days, the cells almost filled the large pores of the scaffold and spread in all directions. However, when the concentration of luteolin increased to 100 μg/ml, most of the cells were detached from the scaffold and the number of cells decreased observably (Fig. 6A). When the concentration increased to 200 μg/ml, the number of cells on the 3D scaffold also decreased significantly (Fig. 6B). We observed that the green fluorescence of the 2D and 3D scaffolds was significantly reduced after drug treatment, and the amount of live cells was also significantly reduced. With the extension of the treatment time, the staining results of the scaffolds at the same drug concentration for 48 h showed a further decrease in the amount of live cells compared to the scaffolds at 24 h. On this basis, the results comparing the drug inhibition in 2D and 3D tumor models were shown in Fig 6. In the presence of the same concentration of luteolin, A375 cells showed greater resistance in the 3D culture model than in the 2D culture model. This is because the drug sensitivity of tumor cells in the 3D tumor model is lower than that of the 2D culture model. As shown in Fig. 6A, after adding the drug to the 2D scaffold, the cells showed a dispersed monolayer distribution, and luteolin could act directly on the cells. The cells on the 3D scaffold showed an aggregated distribution, and the drug could only act directly on a portion of the cells on the surface of the cell mass. The cells inside were exposed to lower concentrations of the drug than the peripheral cells that were directly exposed to the drug. Hypoxia or cell cycle changes in the 3D tumor model may also lead to differences in drug sensitivity [27]. Another reason for this could be the continuous accumulation of A375 cells on the 3D scaffold, forming cell clusters in the pores of the scaffold, which helps to achieve cell-cell and ECM interactions that are closer to the in vivo environment [12]. On the contrary, 2D culture could not form a tumor tissue structure similar to the in vivo environment, which limits the morphology and survival of cells.

CCK-8 assay was used to compare the survival rates of A375 cells cultured in 2D and 3D scaffolds at different drug concentrations. From the results shown in Fig. 6C, it can be noted that luteolin had a significant effect on the proliferation of A375 cells, which is consistent with the results of the live/dead staining method analysis. Figure 6A showed the absorbance of A375 cells after exposure to luteolin (50, 100, 150, 200, 250 μg/ml), measured with CCK-8. In the presence of different concentrations of luteolin, the cell survival rate decreased significantly with increasing time of drug action. The reason was that the inhibitory effect of the drug was relatively small during the first 24 h. After 48 h, the effect of the drug was further enhanced, apoptosis was intensified, and the number of cells was further reduced. A375 cells showed a dose- and time-dependent response to luteolin, and the higher the concentration and the longer the drug action time, the lower the cell survival rate [33, 34]. Fig. 6C also showed that the cell survival rate on 3D-printed scaffolds was higher than 2D- cultured scaffolds at the same drug concentration, which is consistent with the junction analysis described above.

Live/dead staining and CCK-8 assays showed that the inhibitory effect of luteolin on 2D- and 3D-cultured A375 cells was time- and dose-dependent. Although there were differences in drug sensitivity between the two culture systems, it was certain that luteolin has an inhibitory effect on A375 tumor cells.

In summary, the GelMA/PEGDA scaffold produced by 3D bioprinting technology provides a more efficient means for the in vitro culture of A375 tumor cells. We used a combination of extrusion printing and UV light curing. With the addition of a photoinitiator, both GelMA and PEGDA can cure quickly at 365nm light, which is conducive to the stacking of printing layers and the rapid prototyping of 3D structures. The combination of light-curing technology improves the precision of extrusion printing [22]. The mechanical strength, porosity, swelling rate and biocompatibility of GelMA/PEGDA scaffold material were tested to prove that its performance meets the requirements of the study. SEM results showed that the porosity and pore size of the scaffold material are suitable for cell adhesion and growth. The results of CCK-8 and live/dead staining indicated that the 3D scaffold is more suitable for the proliferation of tumor cells. In addition, cell morphology staining results showed that 3D-bioprinted hydrogel scaffolds were more suitable for tumor cell expansion and differentiation compared to 2D culture. Preliminary studies of protein expression in cells on 2D and 3D scaffolds were also performed, showing that cells cultured on 3D-bioprinted scaffolds had higher levels of MMP-9 protein secretion and that the cells exhibited greater aggressiveness. Studies on the effect of luteolin on A375 cells also revealed that the cytotoxicity of luteolin was time- and concentration-dependent. Furthermore, 22.62 ± 1.92% survival was achieved in 2D culture (50 μg/ml, 48 h) and 31.81 ± 2.61% was achieved in 3D culture (50 μg/ml, 48 h). In the 3D culture system, A375 cells were more resistant to the drug. In conclusion, 3D-bioprinted cell mass models offer a new approach for the construction of in vitro tumor models and the screening of anticancer drugs with great potential for further development.
